# ZC4H2 stabilizes Smads to enhance BMP signalling, which is involved in neural development in *Xenopus*

**DOI:** 10.1098/rsob.170122

**Published:** 2017-08-16

**Authors:** Pengcheng Ma, Biyu Ren, Xiangcai Yang, Bin Sun, Xiaoliang Liu, Qinghua Kong, Chaocui Li, Bingyu Mao

**Affiliations:** 1 State Key Laboratory of Genetic Resources and Evolution, Kunming Institute of Zoology, Chinese Academy of Sciences, Kunming 650223, People's Republic of China; 2 Institute of Health Sciences, Anhui University, Hefei 230601, People's Republic of China; 3 Kunming College of Life Science, University of Chinese Academy of Sciences, Kunming 650203, People's Republic of China; 4 Key Laboratory of Animal Models and Human Disease Mechanisms of the Chinese Academy of Sciences and Yunnan Province, Kunming Institute of Zoology, Kunming 650223, People's Republic of China

**Keywords:** ZC4H2, Smad, Smurf, bone morphogenesis protein signalling, neural development

## Abstract

Bone morphogenetic proteins (BMPs) play vital roles in regulating stem cell maintenance, differentiation and embryonic development. Intracellularly, BMP signalling is mediated by Smad proteins, which are regulated post-transcriptionally through reversible phosphorylation and ubiquitination. ZC4H2 is a small nuclear protein associated with intellectual disability and neural development in humans. Here, we report that ZC4H2 is highly expressed in the developing neural system and is involved in neural patterning and BMP signalling in *Xenopus*. Knockdown of ZC4H2 led to expansion of the expression of the pan neural plate marker Sox2 in *Xenopus* embryos. In mammalian cells, ZC4H2 promotes BMP signalling and is involved in BMP regulated myogenic and osteogenic differentiation of mouse myoblast cells. Mechanistically, ZC4H2 binds and stabilizes Smad1 and Smad5 proteins through reducing their association with the Smurf ubiquitin ligases and thus their ubiquitination. We also found that a group of ZC4H2 mutations, which have been isolated in patients with intellectual disorders, showed weaker Smad-stabilizing activity, suggesting that the ZC4H2–Smad interaction might contribute to proper neural development in humans.

## 1. Introduction

Bone morphogenetic proteins (BMPs) belong to the transforming growth factor β (TGFβ) superfamily. They are involved in a broad range of cellular responses in metazoa, including cell proliferation, differentiation and embryonic development [1–4]. In vertebrate embryos, the BMP pathway provides essential signals that induce and pattern the primary germ layers, regulate tissue morphogenesis and left–right asymmetry, and affect cellular pluripotency, differentiation, growth and death [4,5]. In *Xenopus* embryos in particular, mesoderm and endoderm are induced by BMP ligands, acting together with FGFs, and early tissue patterning is achieved by BMPs alongside Wnt and FGF signals. In the ectoderm, different levels of BMP signalling trigger differentiation of the epidermis, neural crest, sensory placodes and neural tissues [5]. To maintain homeostasis in adults, the BMP signal also participates in tissue remodelling and regeneration. BMPs can determine the fate of mesenchymal stem cells by stimulating their differentiation into the chondro-osteoblastic lineage and meanwhile blocking their differentiation into the myoblastic lineage. In response to BMP signals, critical osteogenic transcription factors, such as Runx2 and Ostrix, are induced and drive efficient osteogenic differentiation. On the other hand, BMPs can inhibit myogenic differentiation by suppressing the expression of myogenic basic helix-loop-helix (bHLH) transcriptional factors, such as MyoD and Myf5, and/or inducing the expression of Id (inhibitor of differentiation or inhibitor of DNA binding) proteins that block the DNA-binding ability of bHLH transcriptional factors [2,6–10].

BMP signalling is canonically transferred by the Smad (signalling mothers against decapentaplegic) cascade. BMP ligands can bind to type I and type II receptors on the cell surface. The type II receptors phosphorylate and activate the type I receptors, which in turn phosphorylate downstream receptor-regulated Smads (R-Smads), i.e. Smad1 and Smad5 (or Smad1/5). The activated phospho-R-Smads form complexes with Smad4 and translocate into the nucleus. The Smad complex acts as a transcriptional activator or repressor to regulate target genes expression [2,3,11–13]. In addition to phosphorylation by BMP receptors for their activation, the cytoplasm–nuclear shutting of Smads is also tightly controlled by the phosphorylation and de-phosphorylation cycling [14–18]. Alongside phosphorylation, Smad1/5 are also regulated by the ubiquitination and de-ubiquitination system. The HECT (homologous to the E6-AP carboxyl terminus) type ubiquitin ligases Smurf1 and Smurf2 were first reported to target Smad1/5 for polyubiquitination and proteasomal degradation through direct interaction with their PY motifs in the linker region [1,8,11,19–22]. Several other ubiquitin ligases that target Smads have also been isolated, including SCF/Roc1, NEDD42, WWP1 and HsN3 [20,22–24]. Moreover, non-proteolytic ubiquitination through atypical ubiquitin linkages and monoubiquitination have also been noted in Smad activity regulation [25]. A number of deubiquitination enzymes were also reported to regulate the stability or activity of Smads, such as USP15 and USP9X/FAM [25,26].

ZC4H2 is a zinc-finger protein belonging to the family of proteins with a C-terminal zinc-finger domain characterized by four cysteine residues and two histidine residues. Additionally, ZC4H2 has a coiled-coil domain. Association between ZC4H2 mutation and human intellectual disability syndrome has been reported by two independent studies recently. They both provided evidence that ZC4H2 is specifically expressed in the developing neural system and required for neural development in zebrafish [27,28]. Although the ZC4H2 mutations found in patients are predicted to destabilize the protein and show impaired restoration activity in ZC4H2 mutant or morphant zebrafish embryos, little is known about the underlying molecular mechanism for ZC4H2 in neural development and the related human diseases. In this study, we report that ZC4H2 is a new modulator of BMP signalling, through which ZC4H2 is involved in BMP-mediated neural patterning in *Xenopus* and stem cell differentiation in mammalian cells. Mechanistically, ZC4H2 stabilizes Smads by attenuating their ubiquitination via inhibiting the association between Smurfs and Smads. Furthermore, we also provide evidence to suggest that the impaired Smads-stabilizing activity of ZC4H2 mutants found in patients with intellectual disability might contribute to the related disease progression.

## 2. Results

### 2.1. ZC4H2 is involved in *Xenopus* neural development

**Figure 1.**
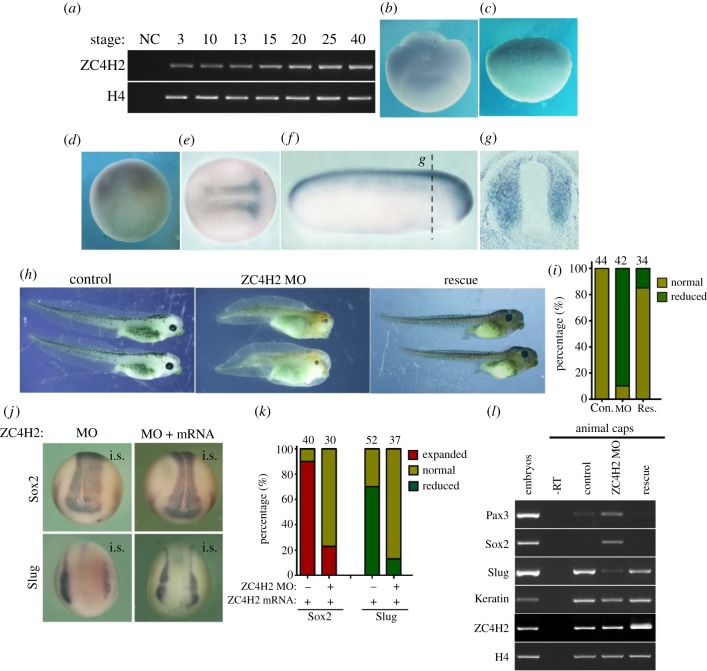
ZC4H2 is required for neural development in *Xenopus*. (*a*) RT-PCR analysis of the developmental expression of ZC4H2 in *Xenopus*. H4 is an internal control. NC, negative control without reverse transcriptase in the RT reaction. (*b–g*) The expression pattern of ZC4H2 revealed by *in situ* hybridization at cleavage embryos (*b,c* lateral view), gastrula embryos (*d*, ventral view), stage 15 (*e*, dorsal view) and stage 25 (*f*, lateral view) *Xenopus* embryos. (*g*) Paraffin sections of the embryos shown in (*e*) showed the expression of ZC4H2 at neural tubes. (*h*) Morphology of tadpoles (stage 42) injected with standard MO (25 ng), ZC4H2 MO (25 ng) and ZC4H2 MO (25 ng) with ZC4H2 mRNA (0.6 ng). Embryos were injected in both blastomeres at the two-cell stage and raised to the indicated tadpole stage. (*i*) Statistics of the data shown in panel (*h*). Con., control, Res., rescue. (*j*) ZC4H2 MO (25 ng) with or without ZC4H2 mRNA (0.6 ng) was injected into one cell of four-cell-stage embryos, and whole-mount *in situ* hybridization with probes of Sox2 and Slug was processed at stage 14–16. GFP mRNA was co-injected to trace the injected sides (on the right sides). i.s., injected side. (*k*) Statistics of the data shown in panel (*j*). (*l*) RT-PCR analysis in animal cap assay showing the effect of ZC4H2 on the expression of the genes of Pax3, Sox2, Slug and Keratin. Embryos were injected in both blastomeres at the two-cell stage and the animal cap tissues were dissected at stage 8 and then were cultured for 20–24 h before harvesting. RT, negative control with reverse-transcriptase omitted in the RT reaction; embryos, RNA template from whole embryos was used as a positive control. H4 is an internal control.

Semi-quantitative RT-PCR was used to investigate the temporal expression patterns of ZC4H2 during *Xenopus* embryonic development. ZC4H2 mRNA was expressed from the early cleavage stages to the swimming tadpole stages that we examined (figure 1*a*). Whole mount *in situ* hybridization was carried out to determine its spatial expression pattern. ZC4H2 transcripts were detected at the animal hemispheres of the cleavage embryos (figure 1*b,c*) and at the dorsal marginal zone of the gastrula embryos (figure 1*d*). As the embryos develop, ZC4H2 transcripts were mainly localized in the developing neural tissues, i.e. neural plate at neural stages and neural tube at tadpode stages (figure 1*e–g*). To determine the potential function of endogenous ZC4H2 during *Xenopus* development, knockdown experiments were carried out with specific morpholinos (MO) against ZC4H2, which efficiently blocked the expression of a GFP reporter carrying its targeted sequences when co-injected into *Xenopus* embryos (data not shown). Interestingly, knockdown of ZC4H2 led to reduction of head and eye structures with shortened trunks and less pigments, an effect that was largely rescued by co-injection of ZC4H2 mRNA (figure 1*h,i*). At the neural stage, the expression of Sox2, a pan neural plate marker, expanded while Slug, a neural crest marker, showed reduced and marginalized expression pattern, and these effects were also well rescued by co-injection of ZC4H2 mRNA (figure 1*j,k*). In *Xenopus* animal caps, Sox2 and Pax3, two neural plate markers, were upregulated while Keratin, an epidermis marker and Slug, a neural crest marker, were downregulated when ZC4H2 was knocked-down, which could all be largely rescued by co-injection with ZC4H2 mRNA (figure 1*l*). These results imply that ZC4H2 is involved in neural development in *Xenopus*.

### 2.2. ZC4H2 regulates BMP signalling in *Xenopus* embryos and C2C12 cells

**Figure 2.**
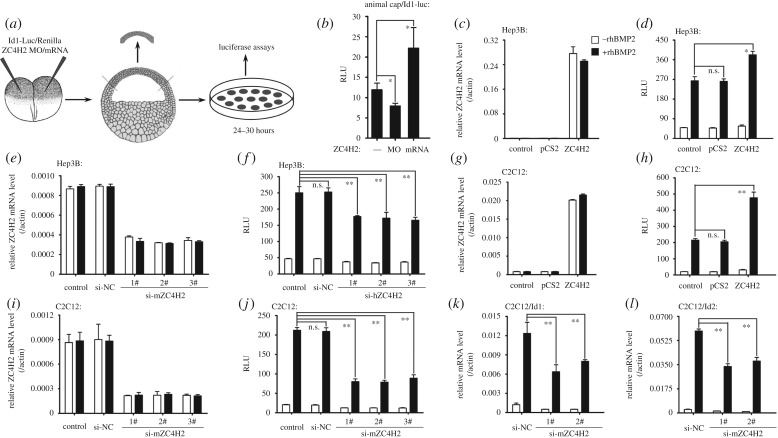
ZC4H2 regulates BMP signalling. (*a,b*) The effect of ZC4H2 knockdown or overexpression on the expression of the BMP reporter Id1-Luc in *Xenopus* animal caps. (*a*) Schematic drawing showing the experimental strategy for reporter assays in *Xenopus* animal caps. Embryos were injected with the indicated MO/mRNAs/plasmids in both blastomeres at the two-cell stage and the animal cap tissues were dissected at stage 8 and then were cultured for 20–24 h before harvesting. RLU, relative light units. *p < 0.05. (*c*–*j*) ZC4H2 over-expression enhanced while knockdown reduced rhBMP2-induced expression level of Id1-Luc reporter in Hep3B (*c–f*) and C2C12 cells (*g–j*). Note that BMP treatment has no effect on either exogenous and endogenous ZC4H2 mRNA levels in both the cell lines (*c,e,g,i*). Hep3B or C2C12 cells were transfected with the indicated plasmids or siRNAs together with Id1-Luc reporter plasmids and then treated with rhBMP2 for 24 h. Luciferase activity was measured at 48 h after transfection. RLU, relative light units. n.s., not significant. *p < 0.05; **p < 0.01. NC, negative control. (*k,l*) ZC4H2 knockdown in C2C12 cells decreases mRNA levels of Id1 (*k*) and Id2 (*l*) induced by rhBMP2. C2C12 cells were transfected with the indicated siRNAs and treated with rhBMP2 for 24 h before harvesting. Total RNA was extracted for real-time PCR analysis at 72 h after transfection. **p < 0.01. NC, negative control.

As *Xenopus* ectoderm is patterned by BMP signalling gradient, we tested whether ZC4H2 is involved in BMP signalling. In BMP luciferase reporter assays in *Xenopus* animal caps, knockdown of ZC4H2 downregulated while its overexpession upregulated the expression level of the BMP reporter Id1-Luc [29] (figure 2*a,b*). Next, we examined the effects of ZC4H2 overexpression or knockdown on BMP signalling in Hep3B and C2C12 cells, two BMP responsive mammalian cell lines. Firstly, we examined ZC4H2 level by real-time RT-PCR in the ZC4H2 overexpressing or knocking-down Hep3B or C2C12 cells and found that all the siRNAs used for targeting human ZC4H2 in Hep3B and mouse ZC4H2 in C2C12 cells showed significant knocking-down efficiency for endogenous ZC4H2 and that both cells received a large amount of exogenous ZC4H2 transcripts when the ZC4H2 plasmids were transfected into them. Also, BMP treatment shows no effects on either the endogenous or exogenous ZC4H2 expression in both the cell lines (figure 2*c,e,g,i*). In both Hep3B and C2C12 cells, the transactivation of Id1-Luc reporter by BMP2 was enhanced by overexpression of ZC4H2, and reduced by three independent siRNA-mediated knockdowns of endogenous ZC4H2 (figure 2*c–j*). The mRNA levels of Id1 and Id2, two established BMP target genes, were decreased when ZC4H2 was knocked-down in C2C12 cells (figure 2*k,l*).

**Figure 3.**
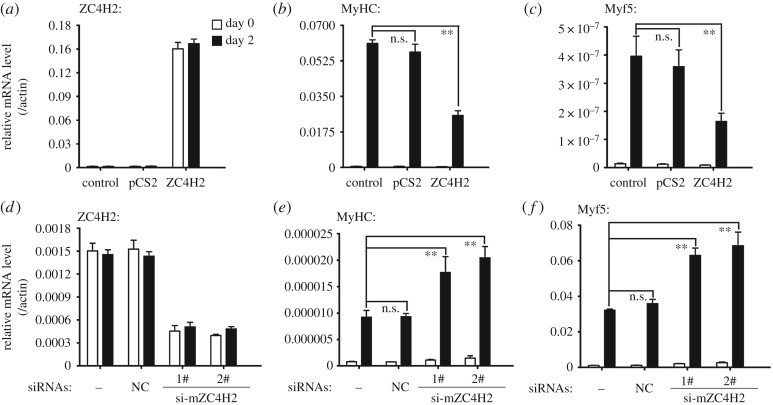
ZC4H2 inhibits myogenic differentiation of C2C12 cells. (*a–c*) mRNA levels of ZC4H2, myogenic markers MyHC and Myf5 during myogenic induction in C2C12 cells with ZC4H2 over-expressed. C2C12 cells were firstly transfected with the indicated plasmids 8 h before myogenic induction in DMEM with the addition of 2% horse serum for 2 days. After induction, total RNAs were isolated and analysed by real-time PCR. (*d–f*) mRNA levels of ZC4H2, myogenic markers MyHC and Myf5 during myogenic induction when ZC4H2 was knocked-down. C2C12 cells were transfected with the indicated siRNAs and then treated with the induction medium for 2 days. The experiments were carried out as described for panel (*a–c*). n.s., not significant; **p < 0.01. NC, negative control.

**Figure 4.**
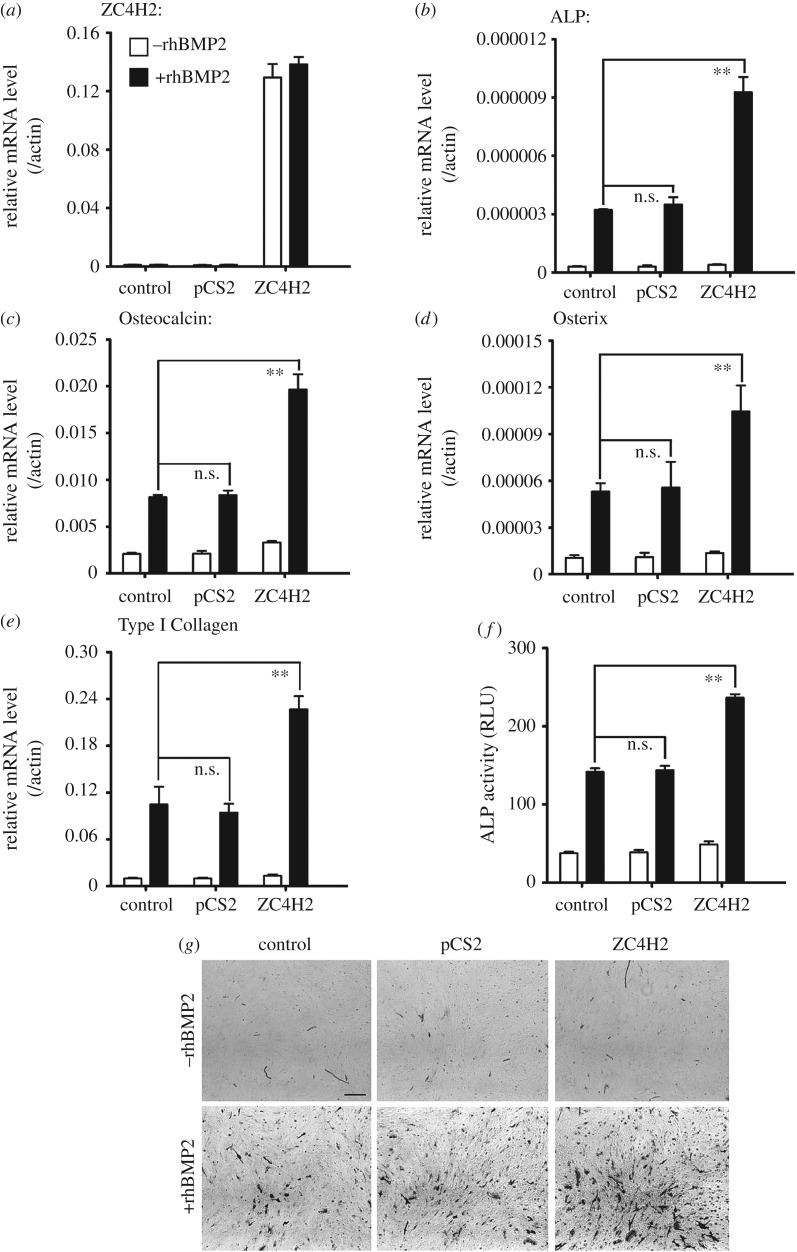
ZC4H2 overexpression enhanced BMP-induced osteogenesis of C2C12 cells. (*a*) Real-time PCR assay showed that ZC4H2 mRNA level in different plasmids transfected C2C12 cells during BMP-induced osteogenesis. C2C12 cells were firstly transfected with the indicated plasmids and then treated with 50 ng ml^−1^ rhBMP2 for 3 day to induce osteogenesis. Then total RNAs were isolated and then real-time PCR assays were carried out. (*b–e*) ZC4H2 overexpression enhanced the expression levels of BMP-induced osteogenic markers ALP (*b*), Osteocalcin (*c*), Osteorix (*d*) and Type I Collegan (*e*). The experiments were carried out as described above for panel (*a*). (*f,g*) ZC4H2 overexpression increased rhBMP induced ALP activity in C2C12 cells. C2C12 cells were treated for osteogenesis as described in panel (*a*). After induction, ALP activity was analysed by a ALP fluorometric kit (*f*) or NBT/BCIP staining (*g*). n.s., not significant; **p < 0.01.

**Figure 5.**
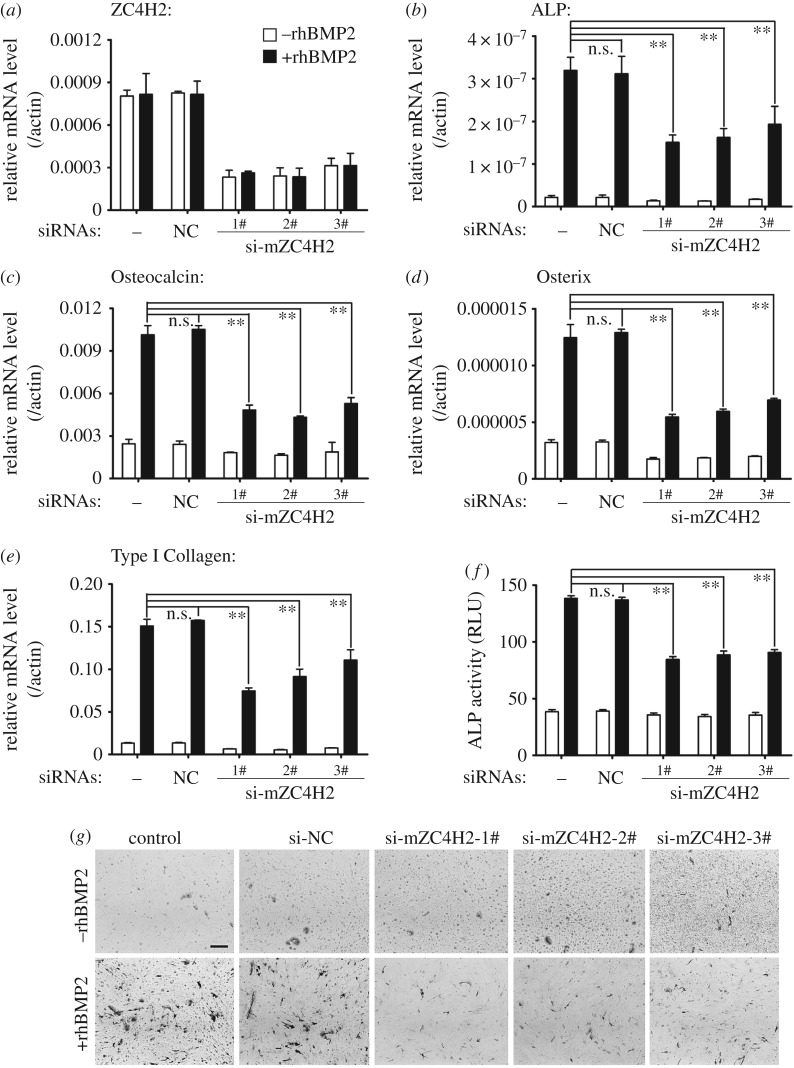
ZC4H2 is required for BMP induced osteogenic differentiation in C2C12 cells. (*a*) ZC4H2 mRNA level in C2C12 cells with different siRNAs transfected during BMP-induced osteogenic differentiation. C2C12 cells were transfected with negative control or three independent siRNAs targeted for ZC4H2 and then were treated with 50 ng ml^−1^ rhBMP2 for 3 days to induce osteogenic differentiation. Then total RNAs were isolated and real-time PCRs were carried out. (*b–e*) ZC4H2 knockdown decreased the expression levels of BMP induced osteogenic markers ALP (*b*), Osteocalcin (*c*), Osterix (*d*) and Type I Collagen (*e*). The experiments were carried out as described above for panel (*a*). (*f,g*) ZC4H2 knockdown reduced rhBMP2-induced ALP activity in C2C12 cells. C2C12 cells were treated for osteogenesis as described above in panel (*a*) after the indicated siRNAs were transfected. After induction, ALP activity was analysed by an ALP fluorometric kit (*f*) or NBT/BCIP staining (*g*). NC, negative control. n.s., not significant; **p < 0.01.

The mouse myoblast cell line C2C12 has been a model system to analyse BMP signalling, in which BMP inhibits its myogenic differentiation while promoting its osteogenic differentiation [6,8]. When cultured in myogenesis induction medium, C2C12 cells typically exhibited induced expression of myogenic indicators, including Myf5 and MyHC (figure 3). Under such a condition, their expression levels were markedly decreased when ZC4H2 was over-expressed while increased when the cells were transfected with ZC4H2 siRNAs (figure 3). When treated with recombinant human BMP2 protein, C2C12 cells quickly undergo osteogenic differentiation and express osteoblast markers such as alkaline phosphatase (ALP), Osteocalcin, Osterix and Type I Collagen (figure 4). Overexpression of ZC4H2 enhanced the expression of the rhBMP2-mediated induction of osteoblast markers (figure 4*b–e*) and increased the ALP activity induced by rhBMP2 treatment (figure 4*f,g*). In contrast, knockdown of ZC4H2 expression by siRNAs reduced BMP-induced expression of the osteoblast markers and ALP activity (figure 5). These observations support a positive role of ZC4H2 in BMP signalling during BMP-regulated myoblast differentiation. Note that the endogenous ZC4H2 level remained the same during myogenic differentiation or BMP-induced osteogenic differentiation of C2C12 cells (figures 3*a,d*, 4*a* and 5*a*).

### 2.3. ZC4H2 stabilizes Smad1 and Smad5

**Figure 6.**
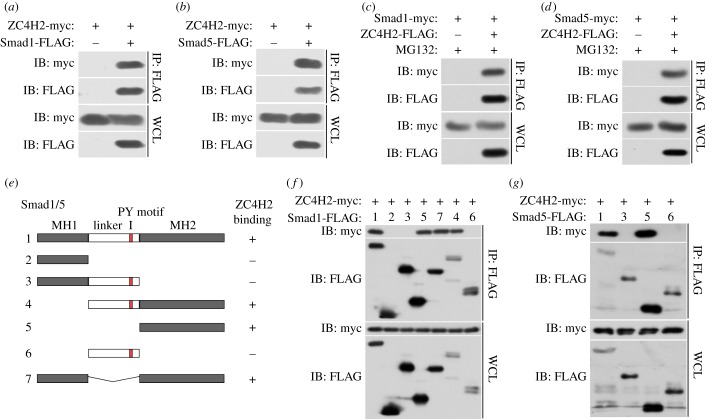
ZC4H2 interacts with Smad1/5. (*a,b*) ZC4H2 was co-immunoprecipitated with Smad1 (*a*) or Smad5 (*b*). (*c,d*) Smad1 (*c*) or Smad5 (*d*) was coimmunoprecipitated with ZC4H2. HEK293 cells were transiently transfected with different combinations of expression vectors of ZC4H2 and Smad1/5, as indicated. Cell lysates were incubated with anti-FLAG beads, washed, and subsequently analysed by western blotting. (*e*) Schematic of the structures of Smad1/5 truncates. (*f,g*) The interaction between ZC4H2 and Smad1 (*f*) or Smad5 (*g*) different truncations. HEK293 cells were transiently transfected with different combinations of expression vectors of myc-tagged ZC4H2 and FLAG-tagged various Smad1 (*e*) or Smad5 (*f*) truncates, as indicated. Cell lysates were incubated with anti-FLAG beads and subsequently analysed by western blotting. WCL, whole-cell lysate; IB, immunoblot; IP, immunoprecipitation.

Because ZC4H2 is predicted as a nuclear protein with a highly conserved nuclear location signal at its C terminus and Smad, the key component of the BMP signalling pathway, can shuttle between cytoplasm and nucleus, we hypothesized that ZC4H2 regulates BMP signalling by directly interacting with Smads. To test this hypothesis, co-immunoprecipitation (co-IP) assays were carried out. In HEK293 cells transiently co-transfected with FLAG-tagged Smad1 or Smad5 and myc-tagged ZC4H2, Smad1 and Smad5 co-immunoprecipitated with ZC4H2 (figure 6*a,b*). In the reverse experiment, ZC4H2 also co-immunoprecipitated with both Smad1 and Smad5 (figure 6*c,d*). To map the domains of Smad proteins that are responsible for the observed interaction, a series of Smad1 and Smad5 truncated constructs were tested via IP assays in HEK293 cells. Smad proteins are approximately 465 amino acids in length and consist of two globular domains (MH1 and MH2 domains) linked by a linker region in between [12]. Our results showed that it is the MH2 domain of Smad1 or Smad5 that is responsible for their interaction with ZC4H2, as all the truncates containing the MH2 domain could pull-down ZC4H2 while the MH2 domain deleted truncates could not (figure 6*e–g*).

**Figure 7.**
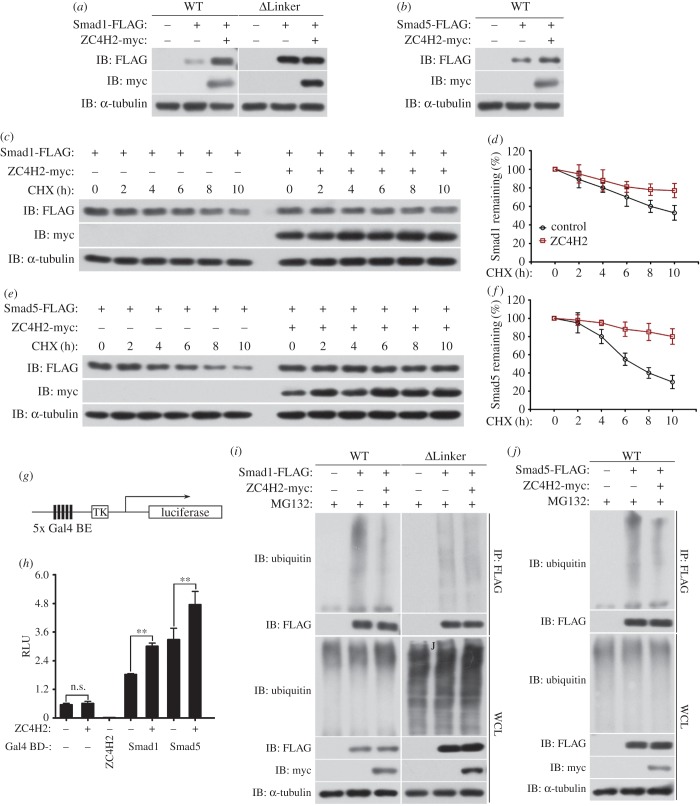
ZC4H2 stabilizes Smad1/5 by attenuating their ubiquitination. (*a,b*) Smad1 and Smad5, but not Smad1-ΔLinker, were stabilized by ZC4H2 overexpression. FLAG-tagged Smad1, Smad5 or Smad1-ΔLinker and myc-tagged ZC4H2 plasmids were transfected into HEK293 cells as indicated. After 48 h, cell lysates were analysed by western blotting. (*c–f*) Effects of ZC4H2 overexpression on the stability of Smad1 (*c,d*) or Smad5 (*e,f*). HEK293 cells were transiently transfected with the indicated plasmids. At 36 h post-transfection, cycloheximide was added to all samples, and the cells were then harvested at the time points indicated. Levels of Smad1 or Smad5 were determined by western blotting with anti-FLAG antibody. In all cases, α-tubulin was used as a loading control. The relative levels of Smad1 or Smad5 were quantified densitometrically and normalized against α-tubulin (*d,f*). The data shown in panel (*c*,*e*) are the averages of three independent experiments. (*g*) Schematic diagram of the pGal4-TK-Luc reporter construct. Gal4 BE, Gal4 binding elements. (*h*) ZC4H2 enhanced the transcriptional activity of Smad1 and Smad5. Gal4-Luc reporter construct was transfected into HEK293 cells together with the combination of myc-tagged ZC4H2 and Gal4 fused Smad1/5 or ZC4H2, as indicated. The luciferase activity was measured at 48 h after transfection. n.s., not significant; **p < 0.01. (*i,j*) Effects of ZC4H2 overexpression on the polyubiquitination status of Smad1, Smad1-ΔLinker (*i*) or Smad5 (*j*) proteins. HEK293 cells were transiently transfected with the indicated plasmids and treated for 8 h with MG132 before harvesting. Smad proteins were coimmunoprecipitated with anti-FLAG antibody and then detected for polyubiquitin chains with the antibody against ubiquitin. WT, wild-type; WCL, whole-cell lysate; IB, immunoblot; IP, immunoprecipitation; CHX, cycloheximide.

Interestingly, we noted that co-transfection of ZC4H2 increased the protein levels of both Smad1 and Smad5 in HEK293 cells, but not the level of the ΔLinker-Smad1 (figure 7*a,b*). We inhibited protein synthesis by cycloheximide and assessed the stability of Smad 1 or Smad5 with or without ZC4H2 co-expression in HEK293 cells. The data showed that both the Smad1 and Smad5 proteins were clearly stabilized in the presence of ZC4H2 (figure 7*c–f*). Consistently, ZC4H2 increased the expression of a Gal4 reporter when co-expressed with Gal4-Smad1or Smad5 in a heterologous luciferase reporter assay (figure 7*g,h*).

As ubiquitination plays a key role in Smad stability regulation, we examined whether ZC4H2 may affect the ubiquitination status. The results showed that co-expression of ZC4H2 strongly reduced the ubiquitination of both Smad1 and Smad5 proteins, but not that of the ΔLinker-Smad1, which is relatively weakly ubiquitinated (figure 7*i,j*). The above data imply that ZC4H2 stabilizes Smad1 and Smad5 by attenuating their polyubiquitination.

**Figure 8.**
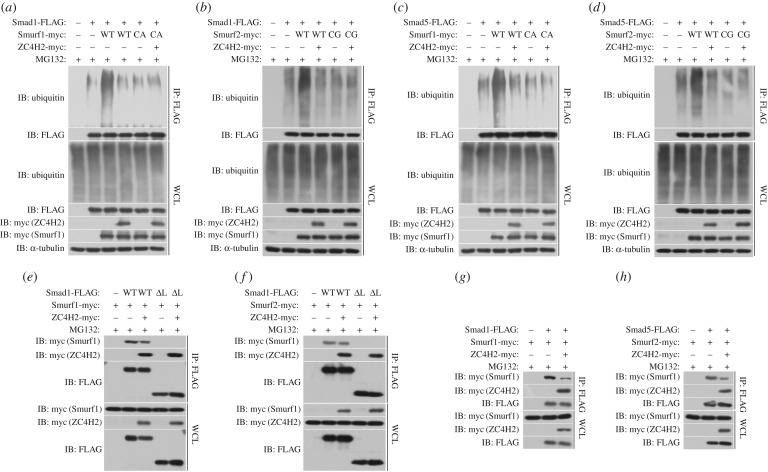
ZC4H2 antagonizes Smurfs activity towards Smads by abolishing the association between Smurfs and Smads. (*a–d*) Effects of ZC4H2 on the polyubiquitination status of Smads mediated by Smurfs. HEK293 cells were transiently transfected with the indicated plasmids and treated for 8 h with MG132 before harvesting. Smad proteins were coimmunoprecipitated with anti-FLAG antibody and then detected for polyubiquitin chains with the antibody against ubiquitin. (*e–h*) Effects of ZC4H2 on the association between Smurfs and Smad1, Smad1-ΔLinker (*e,f*) or Smad5 (*g,h*) proteins. HEK293 cells were transiently transfected with different combinations of expression vectors of ZC4H2 and Smad1, Smad5 or Smad-ΔLinker, as indicated, and treated for 8 h with MG132 before harvesting. Cell lysates were incubated with anti-FLAG beads, washed, and subsequently analysed by western blotting. WCL, whole-cell lysate; IP, immunoprecipitation; IB, immunoblotting; WT, wild-type; ΔL, Smad1-ΔLinker truncates; CA, the ligase dead mutant for Smurf1; CG, the ligase dead mutant for Smurf2.

The most common ubiquitin E3 ligases responsible for Smads ubiquitination are Smurf1 and Smurf2, two typical HECT type E3 ligases. The PY motif in the Linker domain of Smad1 or Smad5 is responsible for the recruitment of Smurfs for the following ubiquitination and degradation [1,8,19–22,30]. A series of *in vitro* ubiquitination assays were carried out to examine the effect of ZC4H2 on Smurf-mediated Smad ubiquitination. Indeed, Smurf1 or Smurf2 dramatically increased the levels of polyubiquitinated Smad1 and Smad5, and this activity was largely inhibited when ZC4H2 was co-expressed (figure 8*a–d*). The ubiquitination level of both Smad1 and Smad5 were not changed when the ligase-dead mutant of Smurf1 (Sumrf1 CA) or Smurf2 (Smurf2 CG) was used, whether ZC4H2 was co-expressed or not (figure 8*a–d*). In co-immunoprecipitation assays, ZC4H2 co-expression reduced the levels of Smurf proteins associated with Smads, while the ΔLinker Smad1 failed to pull-down Smurf1 (figure 8*e–h*), as reported before [1,21]. These data suggest that ZC4H2 reduced the interaction between Smads and Smurfs, and thus their ubiquitination.

### 2.4. ZC4H2 is required for Smad stabilization *in vivo*

**Figure 9.**
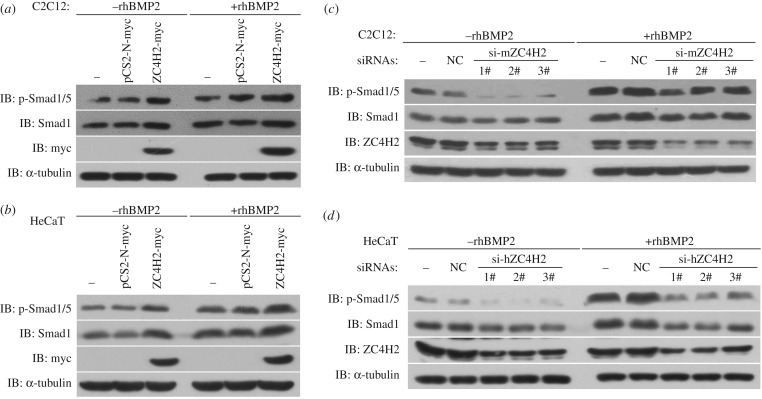
ZC4H2 stabilizes Smad proteins *in vivo*. (*a,b*) ZC4H2 overexpression stabilized Smad proteins in C2C12 (*a*) and HeCaT (*b*) cells. C2C12 or HeCaT cells were transiently transfected with the indicated plasmids and treated with rhBMP2 or not for 24 h before harvesting. Cell lysates were analysed by western blotting. (*c,d*) ZC4H2 knockdown reduced Smad proteins in C2C12 (*c*) and HeCaT (*d*) cells. For knockdown of ZC4H2, three independent siRNAs against mouse or human ZC4H2 were transfected into C2C12 or HeCaT cells, respectively. Cells were treated for 24 h with rhBMP2 and harvested at 72 h after transfection. Cell lysates were analysed by western blotting. NC, negative control; IB, immunoblotting.

Next we examined whether ZC4H2 is involved in the stabilization of endogenous Smads in mammalian cells. In C2C12 and HeCaT cells, the protein levels of both total Smad1 and phosphorylated Smad1/5 were increased when ZC4H2 was over-expressed and were reduced when ZC4H2 was knocked-down by three independent siRNAs, either in the presence or the absence of rhBMP2 treatment (figure 9). These data imply that ZC4H2 is required for Smads stabilization *in vivo*.

**Figure 10.**
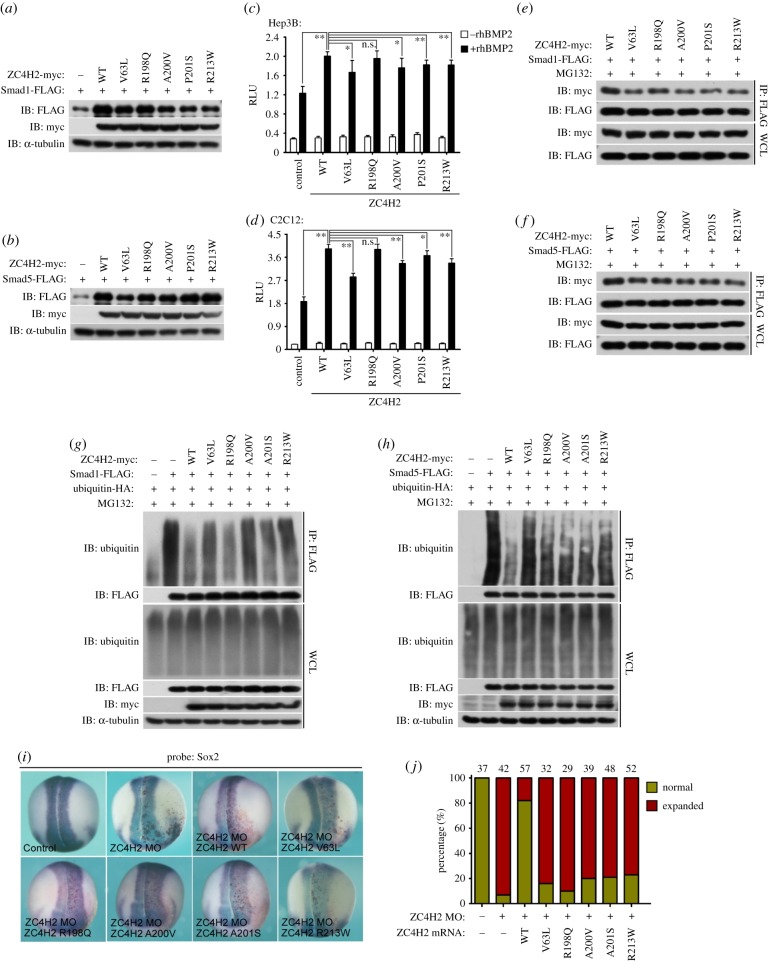
ZC4H2 mutants showed weaker Smad-stabilizing activity and might be involved in neural development in humans. (*a,b*) The effects of overexpression of various ZC4H2 mutants on the protein level of Smad1 (*a*) or Smad5 (*b*) in HEK293 cells. FLAG-tagged Smad1 or Smad5 and myc-tagged various ZC4H2 mutant plasmids were transfected into HEK293 cells as indicated. After 48 h, cell lysates were analysed by western blotting. (*c,d*) The effects of overexpression of various ZC4H2 mutants on the expression of Id1-Luc reporter induced by rhBMP2 treatment in Hep3B (*c*) and C2C12 (*d*) cells. Hep3B or C2C12 cells were transfected with the indicated plasmids together with Id1-Luc reporter plasmids and then treated with rhBMP2 for 24 h. Luciferase activity was measured at 48 h after transfection. RLU, relative light units; n.s., not significant; *p < 0.05 and **p < 0.01. (*e,f*) The association between Smad1 or Smad5 and various ZC4H2 mutants in co-IP assays in HEK293 cells. HEK293 cells were transiently transfected with different combinations of expression vectors of Smad1 or Smad5 and various ZC4H2 mutants as indicated, and treated for 8 h with MG132 before harvesting. Cell lysates were incubated with anti-FLAG beads, washed and subsequently analysed by western blotting. (*g,h*) The effects of overexpression of various ZC4H2 mutants on the polyubiquitination status of Smad1 or Smad5 in HEK293 cells. HEK293 cells were transiently transfected with the indicated plasmids and treated for 8 h with MG132 before harvest. Smad proteins were co-immunoprecipitated with anti-FLAG antibody and then detected for polyubiquitin chains with the antibody against ubiquitin. (*i,j*) *In situ* hybridization assays showed Sox2 expression in the embryos injected with control MO (25 ng), ZC4H2 MO (25 ng) or ZC4H2 MO (25 ng) together with various ZC4H2 mutant mRNAs (0.6 ng). LacZ mRNA was co-injected to trace the injected sides (stained red on the right sides). (*j*) Statistics of the data shown in panel (*i*). WT, wild-type; WCL, whole-cell lysate; IP, immunoprecipitation; IB, immunoblotting.

In humans, a group ofZC4H2 substitutional mutations have been reported to be associated with intellectual disorders, including V63L, A200V, P201S, R198Q and R213 W, which all failed to rescue the ZC4H2 morphants in zebrafish [27,28]. We tested the Smad-stabilizing effects of mutants. Interestingly, we found that all these mutated ZC4H2 proteins showed weaker Smad-stabilizing activity compared to wild-type ZC4H2 proteins (figure 10*a,b*). In the BMP reporter assays, all the ZC4H2 mutants except R198Q mutant proteins showed weaker activity to enhance Smad transactivity (figure 10*c,d*). In addition, we also found that all these mutated ZC4H2 proteins showed weaker interaction with Smad1 and Smad5 in co-IP assays (figure 10*e,f*) and their abilities to prevent Smad1/5 ubiquitination were impaired (figure 10*g,h*). In *Xenopus* embryos, these mutated ZC4H2 constructs also failed to rescue the expansion of Sox2 expression in the ZC4H2 morphants (figure 10*i,j*). We suggest that the weakened Smads-stabilizing activity of the mutated ZC4H2 forms might contribute to the impaired neural development in those patients.

## 3. Discussion

BMP/Smads signalling plays a critical role in ectodermal patterning and counteracts an intrinsic neural induction programme in the nascent ectoderm [4,5]. In this study, we found that ZC4H2 was expressed in the animal hemispheres and ectoderm tissues before/in gastrulation and in the neural specific ectoderm when the embryos established their body axes after gastrulation in developing *Xenopus* embryos. Morpholino-mediated knockdown of ZC4H2 mis-patterned the early ectoderm in *Xenopus* embryos, which was also reproduced in animal caps. The expansion of the neural plate also fits with a potential reduced BMP signalling in the ZC4H2 morphants. We showed that ZC4H2 was involved in BMP signalling in *Xenopus* animal caps, as indicated by changes of expression levels of the Id1-Luc reporter in ZC4H2 knockdown or overexpression experiments. Our work suggests that ZC4H2 might be involved in the BMP signalling mediated neural induction in *Xenopus* embryos. As we provided strong evidence that ZC4H2 stabilizes Smad1/5 and promotes BMP signalling cell autonomously in BMP receiving cells, it is possible that ZC4H2 cell autonomously enhances BMP signalling to facilitate ectoderm differentiation while inhibiting neural induction in *Xenopus* early developing embryos with a broad ectoderm-specific expression pattern.

ZC4H2 is highly conserved across vertebrates. In the ZC4H2 mutant zebrafish larvae, a specific loss of V2 interneurons in the ventral spinal cord and brain was observed [27,28], which implies an important role for ZC4H2 in dorsal–ventral patterning of the spinal cord. Therefore, we hypothesized that ZC4H2 plays two different roles in early and late development embryogenesis stages: it functions as an ectoderm inducer and neural inhibitor through cell autonomous BMP enhancement at early stages with a broader ectoderm expression pattern, and plays an important role in dorsal–ventral patterning of the neural tube at late stages when its expression is mainly restricted to the neural ectoderm tissues. As BMP signalling is also required for dorsal–ventral patterning of spinal cord, we could not rule out the possibility that the cell autonomous BMP enhancing activity of ZC4H2 also contributes to its functions in this late development process ZC4H2 is involved in. And also, whether ZC4H2 is involved in this late neural patterning in *Xenopus* remains to be examined.

Controlling the stability of crucial signalling components via ubiquitination has emerged as an important aspect of BMP signalling regulation. Smurf1 and Smurf2 have been reported as the two most important E3 ligases for BMP signalling regulation by targeting Smads or the membrane receptors, respectively, in a context-dependent manner [1,8,19–22,30–33]. We showed evidence that ZC4H2 modulates BMP signalling by attenuating Smurf-dependent ubiquitination of Smad1/5. It is less likely that BMP membrane receptors are also stabilized by ZC4H2 as: (i) ZC4H2 is predicted as a nuclear protein with a strong nuclear location signal at its C terminus and, in fact, ZC4H2 showed clear nuclear localization in the cells we tested here (data not shown); and (ii) ZC4H2 itself cannot directly interact with Smurfs (data not shown) and only in the presence of wild-type Smads proteins, ZC4H2 and Smurfs existed in the same protein complex and showed Smad stabilizing activity (figure 8). Smad1/5 interact with ZC4H2 and Smurfs through MH2 and PY motifs in Linker domains, respectively (figures 6 and 8). We showed that ZC4H2 reduced the interaction between Smad1/5 and Smurfs. It is possible that interaction with ZC4H2 blocked the surface or destroyed the conformation of Smads needed for Smurfs interaction. And also, it is possible that the lysine residues in Smads targeted by Smurfs were masked by ZC4H2 interaction. We found that a serial ZC4H2 mutation isolated in patients with intellectual disorders showed weaker Smad-stabilizing activity and they all failed to rescue ZC4H2 morphants in *Xenopus*, implying that the ZC4H2–Smad interaction may also contribute to proper neural development in humans [27,28].

BMPs regulate mesenchymal stem cell differentiation through promoting osteogenic differentiation and meanwhile blocking myogenic differentiation. This process is tightly controlled by a range of extracellular, intracellular and nuclear modulators [2,7–9,17,18,34,35]. Using a mouse myoblast C2C12 cell differentiation model, we found that ZC4H2, as a Smads stabilizer, increased the BMP-induced osteogenic and decreased the myogenic potential of C2C12 cells by both gain-of- and loss-of-function approaches. Whether ZC4H2 is involved in muscle/bone development *in vivo* remains to be explored.

## 4. Experimental procedures

### 4.1. *Xenopus* embryos, whole mount *in situ* hybridization, semi-quantitative RT-PCR assays, microinjection and animal cap assays

Adult *Xenopus* *laevis* frogs were obtained from Nasco. *Xenopus* embryos, whole mount *in situ* hybridization, microinjection and animal cap assays were carried out as described previously [36,37]. The sequence of the antisense MO for ZC4H2 which was obtained from Gene Tools is: ZC4H2 MO: 5′-GAATGAAATAAAATGCCAGCAAAAC-3′. MO and mRNA were injected into the dorsal region of 2- to 4-cell stage embryos. In total, 25 ng MO was injected per stitch, 0.6 ng ZC4H2 wild-type or its mutant mRNAs were injected for overexpression or rescue experiments. For *in situ* hybridization, the full-length of ZC4H2 cDNA sequence was used for probe preparation and the probes of Sox2 and Slug were used as described previously. The following primers were used for semi-quantitative RT-PCR assay: *Xenopus* ZC4H2: forward, 5′-ATCAGAAACAAGACCCTCCAAAT-3′ and reverse, 5′-CCTCTGTAGACCCAATGTCATCC-3′; Pax3: forward, 5′-CAGCCGAATTTTGAGGAGCAAAT-3′ and reverse, 5′-GGGCAGGTCTGGTTCGGAGTC-3′; Slug: forward, 5′-T CCCGCACTGAAAATGCCACGATC-3′ and reverse, 5′-CCGTCCTAAAGATGAAGGGTATCCTG-3′; Sox2: forward, 5′-GAGGATGGACACTTATGCCCAC-3′ and reverse, 5′-GGACATGCTGTAGGTAGGCGA-3′; Keratin: forward, 5′-CACCAGAACACAGAGTAC-3′ and reverse, 5′-CAACCTTCCCATCAACCA-3′; and H4 used as loading control: forward, 5′-CGGGATAACATTCAGGGTA-3′ and reverse, 5′-TCCATGGCGGTAACTGTC-3′.

### 4.2. Cell culture, transfection and luciferase reporter assay

HEK293, C2C12, Hep3B and HeCaT cells were maintained in high-glucose Dulbecco's modified Eagle's mediun (DMEM) (Corning) with 10% fetal bovine serum (Gibco). C2C12 cells were cultured in 2% horse serum (Gibco) to induce myogenic differentiation and in 50 ng ml^−1^ rhBMP2 (Pepro Tech) to induce osteoblast-like differentiation. Cells were transfected using Lipofectamine 2000 (Invitrogen) according to the manufacture's instruction. Luciferase reporter assays were carried out as described previously [36–38].

### 4.3. Plasmids and reagents

Full-length mouse ZC4H2 coding region was obtained by PCR according to the sequence in NCBI (NM_001003916.2). Full-length wild-type ZC4H2 and ZC4H2 mutants were cloned into pCS2-C-FLAG/pCS2-N-MYC vectors. Human Smad1, mouse Smad5, Smurf1, Smurf1 CA mutant, Smurf2 and Smurf2 CG mutant plasmids were obtained from Addgene and then were cloned into pCS2-C-FLAG/pCS2-N-MYC vectors. All constructs containing PCR fragments were confirmed by DNA sequencing.

Small interfering RNAs (si-mZC4H2–1#: siG150908075647; si-mZC4H2-2#: siG150908075639 and si-mZC4H2-3#: siG150908075633 for mouse ZC4H2 targeting and si-hZC4H2-1#: siG150908074948; si-hZC4H2-2#: siG150908074958 and si-hZC4H2-3#: siG150908075011 for human ZC4H2 targeting; siN05815122147 for negative control; RioBio) were used to knockdown ZC4H2 in C2C12 and HeCaT cells, respectively.

### 4.4. *In vitro* ubiquitination assay, protein chase assays, immunoprecipitation and western blot analysis

*In vitro* ubiquitination assays, protein chase assays, immunoprecipitation and western blot analysis were carried out as previously described [38,39]. The following antibodies were used for immunoprecipitation or immunoblotting: anti-FLAG (M2, Sigma), anti-myc (M4439, Sigma), anti-α-tubulin (66031, Proteintech), anti-ubiquitin (P4D1, sc-8071, Santa Cruz), anti-Smad1 (385400, Invitrogen), anti-phosphorylated Smad1/5 (9516, Cell Signaling Technology), anti-ZC4H2 (HPA049584, Sigma) and anti-β-actin (ab8827, Abcam).

### 4.5. Reverse transcription and real-time PCR assays

Total RNAs were isolated by using TRNzol reagents (Tiangen). First-strand cDNA was synthesized by using a First-Strand cDNA synthesis kit (Fermantas). Primers for real-time PCR are listed as follows: mouse ZC4H2: forward, 5′-AGCAGGACACAAGGCAGACA-3′ and reverse, 5′-TTGCAAAGAGGGCATATAGG-3′; mouse Id1: forward, 5′-AGAACCGCAAAGTGAGCAAGGT-3′ and reverse, 5′-GGTGGTCCCGACTTCAGACT-3′; mouse Id2: forward, 5′-ATCCCACTATCGTCAGCCTGCAT-3′ and reverse, 5′-ATTCAGATGCCTGCAAGGACAGGA-3′; mouse Myf5: forward, 5′- AGCATTGTGGATCGGATCACGTCT-3′ and reverse, 5′-TGAGTGTCCTTGAGGATGCCTGT-3′; mouse MyHC: forward, 5′- ACGCCATCAGGCTCAAGAAGAAGA-3′ and reverse, 5′-TGAGTGTCCTTGAGGATGCCTTGT-3′; mouse ALP forward, 5′-AGAAGTTCGCTATCTGCCTTGCCT-3′ and reverse, 5′-TGGCCAAAGGGCAATAACTAGGGA-3′; mouse Osteocalcin: forward, 5′-TAGCAGACACCATGAGGACCATCT-3′ and reverse, 5′-CCTGCTTGGACATGAAGGCTTTGT-3′; mouse Osterix: forward, 5′-TCCCTTCTCAAGCACCAATGGACT-3′ and reverse, AAATGAGTGAGGGAA GGGTGGGTA-3′; mouse type I collagen: forward, 5′-TCGGGCCTGCTGGTGTTCGTG-3′ and reverse, 5′-TGGGCGCGGCTGTATGAGTTCTTC-3′; and mouse β-actin: forward, 5′-TGAGCGCAAGTACTCTGTGTGGAT-3′ and reverse, 5′-ACTCATCGTACTCCTGCTTGCTGA-3′.

### 4.6. ALP activity assay and ALP staining

Cells were cultured in 96-well plates, transfected and administered with 50 ng ml^−1^ rhBMP2 for 3 days. ALP activity was examined by using an Alkaline Phosphatase Assay Kit (Fluorometric) (ab83371, Abcam) following the manufacturer's protocols. ALP staining was performed by using BCIP/NBT Alkaline Phosphatase Color Development Kit (C3206, Beyotime) following the manufacturer's instructions.

### 4.7. Statistics

GraphPad and Image J software was used for statistical analysis. Comparisons were performed using the two-tailed Student's *t*-test. *p*-Values of less than 0.05 were considered statistically significant and less than 0.01 were considered statistically very significant. All experiments were carried out at least three times and samples were analysed in triplicate.

## Data accessibility

The datasets supporting this article have been included in the paper.

## References

[1] Zhu H, Kavsak PF, Abdollah S, Abdollah SF, Wrana JL, Wrana JF, Thomsen GH, Thomsen GH 1999 A SMAD ubiquitin ligase targets the BMP pathway and affects embryonic pattern formation. Nature 400, 687–693. (doi:10.1038/23293) 10458166 10.1038/23293

[2] Zhang J, Li L 2005 BMP signaling and stem cell regulation. Dev. Biol. 284, 1–11. (doi:10.1016/j.ydbio.2005.05.009) 15963490 10.1016/j.ydbio.2005.05.009

[3] Bragdon B, Moseychuk O, Saldanha S, King D, Julian J, Nohe A 2011 Bone morphogenetic proteins: a critical review. Cell Signal. 23, 609–620. (doi:10.1016/j.cellsig.2010.10.003) 20959140 10.1016/j.cellsig.2010.10.003

[4] Bier E, De Robertis EM 2015 BMP gradients: a paradigm for morphogen-mediated developmental patterning. Science 348, aaa5838 (doi:10.1126/science.aaa5838) 26113727 10.1126/science.aaa5838

[5] Kuroda H, Wessely O, De Robertis EM 2004 Neural induction in *Xenopus*: requirement for ectodermal and endomesodermal signals via Chordin, Noggin, beta-Catenin, Cerberus. PLoS Biol. 2, E92 (doi:10.1371/journal.pbio.0020092) 15138495 10.1371/journal.pbio.0020092 PMC406387

[6] Katagiri T et al. 1997 Bone morphogenetic protein-2 inhibits terminal differentiation of myogenic cells by suppressing the transcriptional activity of MyoD and myogenin. Exp. Cell Res. 230, 342–351. (doi:10.1006/excr.1996.3432) 9024793 10.1006/excr.1996.3432

[7] Karsenty G, Wagner EF 2002 Reaching a genetic and molecular understanding of skeletal development. Dev. Cell 2, 389–406. (doi:10.1016/S1534-5807(02)00157-0) 11970890 10.1016/s1534-5807(02)00157-0

[8] Ying SX, Hussain ZJ, Zhang YE 2003 Smurf1 facilitates myogenic differentiation and antagonizes the bone morphogenetic protein-2-induced osteoblast conversion by targeting Smad5 for degradation. J. Biol. Chem. 278, 39 029–39 036. (doi:10.1074/jbc.M301193200) 10.1074/jbc.M301193200 PMC3230132 12871975

[9] Yoon BS, Lyons KM 2004 Multiple functions of BMPs in chondrogenesis. J. Cell. Biochem. 93, 93–103. (doi:10.1002/jcb.20211) 15352166 10.1002/jcb.20211

[10] Wu X, Shi W, Cao X 2007 Multiplicity of BMP signaling in skeletal development. Ann. N. Y. Acad. Sci. 1116, 29–49. (doi:10.1196/annals.1402.053) 18083919 10.1196/annals.1402.053

[11] Wrana JL 2000 Regulation of Smad activity. Cell 100, 189–192. (doi:10.1016/S0092-8674(00)81556-1) 10660041 10.1016/s0092-8674(00)81556-1

[12] Massague J, Seoane JF, Wotton D, Wotton D 2005 Smad transcription factors. Genes Dev. 19, 2783–2810. (doi:10.1101/gad.1350705) 16322555 10.1101/gad.1350705

[13] Brazil DP, Church RH, Surae S, Godson C, Martin F 2015 BMP signalling: agony and antagony in the family. Trends Cell Biol. 25, 249–264. (doi:10.1016/j.tcb.2014.12.004) 25592806 10.1016/j.tcb.2014.12.004

[14] Lin X et al. 2006 PPM1A functions as a Smad phosphatase to terminate TGFbeta signaling. Cell 125, 915–928. (doi:10.1016/j.cell.2006.03.044) 16751101 10.1016/j.cell.2006.03.044 PMC6309366

[15] Dai F, Lin X, Chang C, Feng XH 2009 Nuclear export of Smad2 and Smad3 by RanBP3 facilitates termination of TGF-beta signaling. Dev. Cell 16, 345–357. (doi:10.1016/j.devcel.2009.01.022) 19289081 10.1016/j.devcel.2009.01.022 PMC2676691

[16] Bruce DL, Sapkota GP 2012 Phosphatases in SMAD regulation. FEBS Lett. 586, 1897–1905. (doi:10.1016/j.febslet.2012.02.001) 22576046 10.1016/j.febslet.2012.02.001

[17] Zhao Y, Xiao M, Sun B, Zhang Z, Shen T, Duan X, Yu PB, Feng XH, Lin X 2014 C-terminal domain (CTD) small phosphatase-like 2 modulates the canonical bone morphogenetic protein (BMP) signaling and mesenchymal differentiation via Smad dephosphorylation. J. Biol. Chem. 289, 26 441–26 450. (doi:10.1074/jbc.M114.568964) 10.1074/jbc.M114.568964 PMC4176200 25100727

[18] Chen F et al. 2015 Nuclear export of Smads by RanBP3L regulates bone morphogenetic protein signaling and mesenchymal stem cell differentiation. Mol. Cell. Biol. 35, 1700–1711. (doi:10.1128/MCB.00121-15) 25755279 10.1128/MCB.00121-15 PMC4405638

[19] Lin X, Liang M, Feng XH 2000 Smurf2 is a ubiquitin E3 ligase mediating proteasome-dependent degradation of Smad2 in transforming growth factor-beta signaling. J. Biol. Chem. 275, 36 818–36 822. (doi:10.1074/jbc.C000580200) 10.1074/jbc.C000580200 11016919

[20] Gruendler C, Lin YF, Farley J, Farley JF, Wang T 2001 Proteasomal degradation of Smad1 induced by bone morphogenetic proteins. J. Biol. Chem. 276, 46 533–46 543. (doi:10.1074/jbc.M105500200) 10.1074/jbc.M105500200 11571290

[21] Zhang Y, Chang C, Gehling DJ, Gehling DJ, Hemmati-Brivanlou A, Hemmati-Brivanlou A, Derynck R 2001 Regulation of Smad degradation and activity by Smurf2, an E3 ubiquitin ligase. Proc. Natl Acad. Sci. USA 98, 974–979. (doi:10.1073/pnas.98.3.974) 11158580 10.1073/pnas.98.3.974 PMC14694

[22] Izzi L, Attisano L 2004 Regulation of the TGFbeta signalling pathway by ubiquitin-mediated degradation. Oncogene 23, 2071–2078. (doi:10.1038/sj.onc.1207412) 15021894 10.1038/sj.onc.1207412

[23] Komuro A, Imamura T, Saitoh M, Yoshida Y, Yamori T, Miyazono K, Miyazawa K 2004 Negative regulation of transforming growth factor-beta (TGF-beta) signaling by WW domain-containing protein 1 (WWP1). Oncogene 23, 6914–6923. (doi:10.1038/sj.onc.1207885) 15221015 10.1038/sj.onc.1207885

[24] Kim BG, Lee J, Yasuda J, Yasuda J, Ryoo H-M, Cho J-Y 2011 Phospho-Smad1 modulation by nedd4 E3 ligase in BMP/TGF-beta signaling. J. Bone Miner. Res. 26, 1411–1424. (doi:10.1002/jbmr.348) 21308777 10.1002/jbmr.348

[25] Dupont S et al. 2009 FAM/USP9x, a deubiquitinating enzyme essential for TGFbeta signaling, controls Smad4 monoubiquitination. Cell 136, 123–135. (doi:10.1016/j.cell.2008.10.051) 19135894 10.1016/j.cell.2008.10.051

[26] Inui M et al. 2011 USP15 is a deubiquitylating enzyme for receptor-activated SMADs. Nat. Cell Biol. 13, 1368–1375. (doi:10.1038/ncb2346) 21947082 10.1038/ncb2346

[27] Hirata H et al. 2013 ZC4H2 mutations are associated with arthrogryposis multiplex congenita and intellectual disability through impairment of central and peripheral synaptic plasticity. Am. J. Hum. Genet. 92, 681–695. (doi:10.1016/j.ajhg.2013.03.021) 23623388 10.1016/j.ajhg.2013.03.021 PMC3644645

[28] May M et al. 2015 ZC4H2, an XLID gene, is required for the generation of a specific subset of CNS interneurons. Hum. Mol. Genet. 24, 4848–4861. (doi:10.1093/hmg/ddv208) 26056227 10.1093/hmg/ddv208 PMC4527488

[29] Korchynskyi O, ten Dijke P 2002 Identification and functional characterization of distinct critically important bone morphogenetic protein-specific response elements in the Id1 promoter. J. Biol. Chem. 277, 4883–4891. (doi:10.1074/jbc.M111023200) 11729207 10.1074/jbc.M111023200

[30] Lonn P, Moren A, Raja E, Dahl M, Moustakas A 2009 Regulating the stability of TGFbeta receptors and Smads. Cell. Res. 19, 21–35. (doi:10.1038/cr.2008.308) 19030025 10.1038/cr.2008.308

[31] Kavsak P et al. 2000 Smad7 binds to Smurf2 to form an E3 ubiquitin ligase that targets the TGF beta receptor for degradation. Mol. Cell 6, 1365–1375. (doi:10.1016/S1097-2765(00)00134-9) 11163210 10.1016/s1097-2765(00)00134-9

[32] Ebisawa T, Fukuchi M, Murakami G, Chiba T, Tanaka K, Imamura T, Miyazono K 2001 Smurf1 interacts with transforming growth factor-beta type I receptor through Smad7 and induces receptor degradation. J. Biol. Chem. 276, 12 477–12 480. (doi:10.1074/jbc.C100008200) 11278251 10.1074/jbc.C100008200

[33] Balemans W, Van HW 2002 Extracellular regulation of BMP signaling in vertebrates: a cocktail of modulators. Dev. Biol. 250, 231–250. (doi:10.1006/dbio.2002.0779) 12376100

[34] Cao Y, Wang C, Zhang X, Xing G, Lu K, Gu Y, He F, Zhang L 2014 Selective small molecule compounds increase BMP-2 responsiveness by inhibiting Smurf1-mediated Smad1/5 degradation. Sci. Rep. 4, 4965 (doi:10.1038/srep04965) 24828823 10.1038/srep04965 PMC4021816

[35] Zhang Y, Wang C, Cao Y, Gu Y, Zhang L 2016 Selective compounds enhance osteoblastic activity by targeting HECT domain of ubiquitin ligase Smurf1. Oncotarget 7, e10648 (doi:10.18632/oncotarget.10648) 10.18632/oncotarget.10648 PMC5584161 28881580

[36] Ma P, Zhao S, Zeng W, Yang Q, Li C, Lv X, Zhou Q, Mao B 2011 *Xenopus* Dbx2 is involved in primary neurogenesis and early neural plate patterning. Biochem. Biophys. Res. Commun. 412, 170–174. (doi:10.1016/j.bbrc.2011.07.068) 21806971 10.1016/j.bbrc.2011.07.068

[37] Zhang Z, Shi Y, Zhao S, Li J, Li C, Mao B 2014 *Xenopus* Nkx6.3 is a neural plate border specifier required for neural crest development. PLoS ONE 12, e115165 (doi:10.1371/journal.pone.0115165) 10.1371/journal.pone.0115165 PMC4274032 25531524

[38] Kong Q, Zeng W, Wu J, Hu W, Li C, Mao B 2010 RNF220, an E3 ubiquitin ligase that targets Sin3B for ubiquitination. Biochem. Biophys. Res. Commun. 393, 708–713. (doi:10.1016/j.bbrc.2010.02.066) 20170641 10.1016/j.bbrc.2010.02.066

[39] Liu X, Yang X, Li Y, Zhao S, Li C, Ma P, Mao B 2016 Trip12 is an E3 ubiquitin ligase for USP7/HAUSP involved in the DNA damage response. FEBS Lett. 590, 4213–4222. (doi:10.1002/1873-3468.12471) 27800609 10.1002/1873-3468.12471

